# Telephone-based assessment of multiple sclerosis patients at Ain Shams University Hospital in the coronavirus disease 2019 pandemic

**DOI:** 10.1186/s41983-021-00316-1

**Published:** 2021-05-30

**Authors:** Mohamed A. Abdel Hafeez, Dina A. Zamzam, Mahmoud S. Swelam, Alaa Abo Steit, Janet Masoud, Azza Abdel Nasser, Ahmed Hazzou, Eman Hamid, Hany Aref, Magd F. Zakaria, Mohamed M. Fouad

**Affiliations:** Neurology Department, Ain Shams University, Cairo, Egypt

**Keywords:** COVID-19, Multiple sclerosis, Telephone calls, Hauser ambulation index, Multiple sclerosis neuropsychology questionnaire, Symptoms of multiple sclerosis scale

## Abstract

**Background:**

Assessment of multiple sclerosis (MS) patients during the era of the coronavirus disease 2019 (COVID-19) pandemic was confronted with the overwhelmed healthcare facilities in Egypt and fear of the patients to get infected while attending the follow-up visits. This study aimed to assess the value of telephone-based assessments in the follow-up of MS patients. It includes one hundred and five patients who participated in the study and completed 3 telephone-based assessments which are the Hauser Ambulation index, Multiple Sclerosis Neuropsychology Questionnaire (MSNQ), and Symptoms of Multiple Sclerosis Scale (SMSS).

**Results:**

The Hauser Ambulation index was significantly correlated with the latest Expanded Disability Status Scale (EDSS) score done within 1 month from the telephone call (*r*=0.738, *P*<0.001). The analysis of MSNQ scores showed that one-third of the study population had evidence of cognitive and/or neuropsychological impairment. Post hoc analysis regarding the cognitive and psychological impairment component of SMSS revealed that the patients who answered “Never” had significantly lower MSNQ scores compared to those who answered “Sometimes” (*P*=0.016), “Often” (*P*=0.022), and “Always” (*P*=0.001). The comparison of the EDSS scores of the patients regarding the sensory-motor impairment component of SMSS showed a non-significant difference.

**Conclusion:**

The Hauser Ambulation index may be a reliable telephone-based tool for the assessment of physical disability. The MSNQ and the cognitive and psychological impairment component of SMSS can be used for the assessment of cognitive and psychological impairment among patients with MS.

## Background

Coronavirus disease 2019 (COVID-19) is caused by severe acute respiratory syndrome coronavirus 2 (SARS-CoV-2) and has become a global pandemic that overwhelmed most of the healthcare facilities. People with multiple sclerosis (MS), an inflammatory demyelinating and neurodegenerative disorder treated with immune-modulatory disease-modifying therapies (DMTs), need close monitoring during the pandemic of COVID-19 [1]. Moreover, many of the MS patients were unable to access their regular medical services (infusions, follow-up visits, physical and occupational therapy) because of either preoccupation of healthcare facilities or their fear of transportation during the pandemic. This subsequently led to increased implementation of an alternative method of communication as telephone call-based interviews.

Hence, this study aimed to evaluate the value of three telephone-based assessments (Hauser Ambulation index, Multiple Sclerosis Neuropsychology Questionnaire, and Symptoms of Multiple Sclerosis Scale) replacing the routine follow-up visits as a way to monitor patients with multiple sclerosis by telephone and to minimize the hospital visits to decrease the risk of infection.

## Methods

This was a cross-sectional, telephone-based interview study. Patients were recruited from the MS unit at Ain Shams University Hospital. All of the patients who attended the MS unit for follow-up visits, dispensing their medications or receiving their infusion therapies during the period from April 2020 to August 2020, were offered to participate in the study. One hundred and six patients with the diagnosis of MS were recruited to the study after giving their informed consent to participate in the study and agreeing to use their demographic and clinical data in clinical research. Patients were included only if above 18 years and patients with other comorbid neurological diseases or chronic medical illness were excluded.

The records of each patient included demographic data, medical history, key episodes in the course of MS, and Expanded Disability Status Scale (EDSS) scores done at their previous physical visits. Six of the staff members of the MS unit who participated in the study were randomly assigned to a total number of 106 patients and did the telephone-based assessments by themselves.

A telephone call was done for all the patients within 30 days from their latest physical visit to the MS unit. At the beginning of the call, each patient was questioned about any new symptoms that may be suggestive of new relapse since the last visit, and the patient who accounted on symptoms that might be suggestive of new relapse was excluded and advised to have a visit for further assessment. One patient was excluded for that reason, and the study population included 105 patients.

The first scale to be assessed was the Hauser Ambulation index. The patient was questioned about the ambulation distance that can be walked independently, or the need of walking aid or wheelchair during daily activities. And the patients scored as in Table 1 [2].

**Table 1:** Hauser Ambulation index scoring

0 = Asymptomatic; fully active	
1 = Walks normally, but reports fatigue that interferes with athletic or other demanding activities	
2 = Abnormal gait or episodic imbalance; gait disorder is noticed by family and friends; able to walk 25 ft (8 m) in 10 s or less	
3 = Walks independently; able to walk 25 ft in 20 s or less	
4 = Requires unilateral support (cane or single crutch) to walk; walks 25 ft in 20 s or less	
5 = Requires bilateral support (canes, crutches, or walker) and walks 25 ft in 20 s or less; or requires unilateral support but needs more than 20 s to walk 25 ft	
6 = Requires bilateral support and more than 20 s to walk 25 ft; may use wheelchair* on occasion	
7 = Walking limited to several steps with bilateral support; unable to walk 25 ft; may use wheelchair* for most activities	
8 = Restricted to wheelchair; able to transfer self independently	
9 = Restricted to wheelchair; unable to transfer self independently	

After that, the patient was questioned about the 15 self-report items of the Multiple Sclerosis Neuropsychology Questionnaire (MSNQ) which is a self-report screening test that can measure neuropsychological competence and cognitive functioning in several domains including attention and processing speed and memory during activities of daily living in MS patients. If a patient had a score of 27 or more, it was assumed that cognitive impairment and/or neuropsychological problems were present [3].

Lastly, the patient was questioned about the Symptoms of Multiple Sclerosis Scale (SMSS) which is a psychometric scale that is composed of 12 questions that assess multiple symptoms experienced by MS patients. This scale assesses 3 components: bodily dysfunction, cognitive and psychological impairment, and sensory-motor impairment [4].

### Statistical methods

Data entry, processing, and statistical analysis were carried out using SPSS, version 25 (IBM, San, Francisco, CA, USA). The descriptive data were described as frequency and percentage values and median and range for qualitative data and not normally distributed quantitative data. Spearman correlation coefficient was used to find the correlation between 2 or more quantitative non-normally distributed variables; chi-square was used for comparing quantitative variables. For qualitative data, the chi-square test for independent groups was used.

## Results

The study population included one hundred and five patients, seventy-six female (72.4%) and 29 males (27.6%). Relapsing-remitting multiple sclerosis (RRMS) was the most common (90.5% of the study population), the EDSS score done at the latest physical visit before enrollment in the study ranging from 0 to 7 with a median score of 2.5. Table 2 shows the demographic and clinical characteristics data of our patients.

**Table 2:** Demographic and clinical characteristics of the study population

		Number (percentage)
**Age (years)**	**Range**	18–58
	**Median**	34
**Gender**	**Male**	29 (27.6%)
	**Female**	76 (72.4%)
**Type of MS**	**RRMS**	95 (90.5%)
	**SPMS**	9 (8.5%)
	**PPMS**	1 (1.0%)
**Total duration of illness (months)**	**Range**	1–25
	**Median**	5
**EDSS score**	**Range**	0–7
	**Median**	2.5
**EDSS grading**	**Mild disability (0–3)**	69 (65.7%)
	**Moderate disability (3.5–6)**	28 (26.7%)
	**Severe disability (˃6)**	8 (7.6%)
**Number of relapses during the last year**	**No relapse**	40 (38.1%)
	**One relapse**	49 (46.7%)
	**Two relapses**	13 (12.4%)
	**More than two relapses**	3 (2.8%)
**DMT**	**Interferon beta**	49 (46.7%)
	**Dimethyl fumarate**	1 (1.0%)
	**Teriflunomide**	5 (4.7%)
	**Fingolimod**	39 (37.1%)
	**Rituximab**	10 (9.5%)
	**Ocrelizumab**	1 (1.0%)
**Hauser Ambulation index**	**Range**	0–9
	**Median**	1

The scores of the Hauser Ambulation index among the study population ranged from 0 to 9 with a median score of 1. It was found that the Hauser Ambulation index was significantly correlated with the latest EDSS score (*r*=0.738, *P*<0.001).

The scores of MSNQ among the study population ranged from 0 to 58 with a median score of 23. Seventy patients (66.7%) [group A] had a score less than 27 and appeared to have neither cognitive nor neuropsychological problems, while 35 patients (33.3%) [group B] had a score of 27 or more proving to have an evidence of cognitive and/or neuropsychological impairment (Table 3).

**Table 3:** Multiple Sclerosis Neuropsychological Questionnaire (MSNQ)

	Multiple Sclerosis Neuropsychological Questionnaire				
	Never, does not occur (score=0)	Very rarely, no problem (score=1)	Occasionally seldom a problem (score=2)	Quite often, interfere with life (score=3)	Very often, very disruptive (score=4)
	Frequency (%)	Frequency (%)	Frequency (%)	Frequency (%)	Frequency (%)
**Easily distracted**	25 (23.8%)	27 (25.7%)	23 (21.9%)	16 (15.2%)	14 (13.3%)
**Lose thoughts while listening to somebody**	28 (26.7%)	24 (22.9%)	22 (21.0%)	19 (18.1%)	12 (11.4%)
**Slow when try to solve problem**	28 (26.7%)	24 (22.9%)	29 (27.6%)	18 (17.1%)	6 (5.7%)
**Forget appointments**	32 (30.5%)	25 (23.8%)	24 (22.9%)	15 (14.3%)	9 (8.6%)
**Forget what is read**	26 (24.8%)	24 (22.9%)	32 (30.5%)	17 (16.2%)	6 (5.7%)
**Trouble describe show recently watched**	33 (31.4%)	30 (28.6%)	26 (24.8%)	12 (11.4%)	4 (3.8%)
**Instruction repeated**	28 (26.7%)	31 (29.5%)	32 (30.5%)	10 (9.5%)	4 (3.8%)
**Remind to do task**	29 (27.6%)	26 (24.8%)	28 (26.7%)	15 (14.3%)	7 (6.7%)
**Forget errands that were planned**	39 (37.1%)	22 (21.0%)	25 (23.8%)	11 (10.5%)	8 (7.6%)
**Difficulty answering questions**	39 (37.1%)	27 (25.7%)	28 (26.7%)	7 (6.7%)	4 (3.8%)
**Difficulty keeping track of two things at once**	25 (23.8%)	24 (22.9%)	27(25.7%)	22 (21.0%)	7 (6.7%)
**Miss point of what someone say**	33 (31.4%)	25 (23.8%)	24 (22.9%)	17 (16.2%)	6 (5.7%)
**Difficulty control impulse**	23 (21.9%)	24 (22.9%)	22 (21.0%)	20 (19.0%)	16 (15.2%)
**Laugh or cry with little cause**	23 (21.9%)	13 (12.4%)	21 (20.0%)	25 (23.8%)	23(21.9%)
**Talk excessively or focus too much on own interests**	27 (25.7%)	22 (21.0%)	30 (28.6%)	17 (16.2%)	9 (8.6%)
**Total MSNQ score**	**Less than 27** **(group A)**	70		66.7%	
	**27 or more** **(group B)**	35		33.3%	
**Total MSNQ score: median, minimum, maximum**	23	0		58	

The comparison between group A and group B showed non-significant differences as regards age, gender, clinical features of the disease, or disability (Table 4).

**Table 4:** Comparison among study population according to the MSNQ score

		Group A (score in MSNQ ˂27) (***n***=70)	Group B (score in MSNQ ≥27) (***n***=35)	***P***
**Age (years)**	**Range**	18–42	21–58	0.673
	**Median**	33	35	
**Gender**	**Male**	22	7	0.217
	**Female**	48	28	
**Type of MS**	**RRMS**	64	31	0.600
	**SPMS**	5	4	
	**PPMS**	1	0	
**Total duration of illness (months)**	**Range**	24	21	0.700
	**Median**	3	5	
**EDSS score**	**Range**	0–7	0–7	0.383
	**Median**	2.0	3.0	
**EDSS grading**	**Mild (0–3)**	48	21	0.681
	**Moderate (3.5–6)**	17	11	
	**Severe (˃6)**	5	3	
**Number of relapses during the last year**	**No relapse**	28	14	0.568
	**One relapse**	31	18	
	**Two relapses**	11	2	
	**More than two relapses**	2	1	

Table 5 illustrates the results of the components of the Symptoms of Multiple Sclerosis Scale (SMSS).

**Table 5:** Symptoms of Multiple Sclerosis Scale (SMSS)

Symptoms of Multiple Sclerosis Scale (SMSS)		Never	Rarely	Sometimes	Often	Always
		Frequency (%)	Frequency (%)	Frequency (%)	Frequency (%)	Frequency (%)
**Bodily dysfunction**	**Bladder difficulties**	27 (25.7%)	11 (10.5%)	45 (42.9%)	19 (18.1%)	3 (2.9%)
	**Bowel difficulties**	44 (41.9%)	18 (17.1%)	28 (26.7%)	12 (11.4%)	3 (2.9%)
	**Loss of balance**	21 (20.0%)	9 (8.6%)	56 (53.3%)	13 (12.4%)	6 (5.7%)
	**Spasticity**	20 (19.0%)	7 (6.7%)	61 (58.1%)	14 (13.3%)	3 (2.9%)
**Frequency (%)**		112 (26.7%)	45 (10.7%)	190 (45.2%)	58 (13.8%)	15 (3.6%)
**Average**		28	11.25	47.5	14.5	3.75
**Cognitive and psychological impairment**	**Fatigue**	7 (6.7%)	13 (12.4%)	33 (31.4%)	37 (35.2%)	15 (14.3%)
	**Lack of concentration**	15 (14.3%)	8 (7.6%)	44 (41.9%)	26 (24.8%)	12 (11.4%)
	**Inability to communicate**	25 (23.8%)	6 (5.7%)	68 (64.8%)	6 (5.7%)	0 (0.0%)
	**Visual impairment**	23 (21.9%)	7 (6.7%)	53 (50.5%)	19 (18.1%)	3 (2.9%)
**Frequency (%)**		70 (16.7%)	34 (8.1%)	198 (47.1%)	88 (21%)	30 (7.1%)
**Average**		17.5	8.5	49.5	22	7.5
**Sensory-motor impairment**	**Numbness**	22 (21.0%)	14 (13.3%)	44 (41.9%)	20 (19%)	5 (4.8%)
	**Tremors**	20 (19.0%)	7 (6.7%)	61 (58.1%)	14 (13.3%)	3 (2.9%)
	**Pain**	21 (20.0%)	3 (2.9%)	50 (47.6%)	29 (27.6%)	2 (1.9%)
	**Paralysis**	20 (19.0%)	7 (6.7%)	49 (46.7%)	23 (21.9%)	6 (5.7%)
**Frequency (%)**		83 (19.8%)	31 (7.4%)	204 (48.6%)	86 (20.5%)	16 (3.8%)
**Average**		20.75	7.75	51	21.5	4

Post hoc analysis regarding the bodily dysfunction component of SMSS revealed that the patients who answered “Always” had significantly lower EDSS scores compared to those who answered “Sometimes” (*P*=0.045) (Table 6).

**Table 6:** Comparison of EDSS scores in the bodily dysfunction component of SMSS

Bodily dysfunction component of SMSS	EDSS			One-way ANOVA	
	Mean ± SD	Median	Range	***f***	***P***
**Never**	2.95±1.88	2.5	0–5	2.836	0.026
**Rarely**	3.18±1.43	3.5	1–6		
**Sometimes**	2.69±1.73	2.0	0–7		
**Often**	3.55±2.11	3.0	1–7		
**Always**	4.33±2.09	3.5	2–7		
**Post hoc**	**Sometimes versus always**				
**Mean difference**	−1.63462				
**Significance**	0.045				

The comparison of the EDSS scores of the patients regarding the sensory-motor impairment component of SMSS showed a non-significant difference between them (Table 7).

**Table 7:** Comparison of EDSS scores in the sensory-motor impairment component of SMSS

Sensory-motor impairment component of SMSS	EDSS			One-way ANOVA	
	Mean ± SD	Median	Range	***f***	***P***
**Never**	2.830±1.75	2.0	0–5	1.786	0.133
**Rarely**	3.37±1.69	3.0	1–7		
**Sometimes**	2.63±1.56	2.0	0–7		
**Often**	3.23±1.88	2.5	1–7		
**Always**	3.65±2.02	3.5	2–7		

Post hoc analysis regarding the cognitive and psychological impairment component of SMSS revealed that the patients who answered “Never” had significantly lower MSNQ scores compared to those who answered “Sometimes” (*P*=0.016), “Often” (*P*=0.022), and “Always” (*P*=0.001) (Table 8).

**Table 8:** Comparison of MSNQ scores in the cognitive and psychological impairment component of SMSS

Cognitive and psychological impairment component of SMSS	MSNQ	One-way ANOVA	
	Mean ± SD	***f***	***P***
**Never**	17.23±12.01	4.944	0.001
**Rarely**	23.80±9.94		
**Sometimes**	24.93±11.76		
**Often**	25.85±12.82		
**Always**	32.51±12.13		
**Post hoc**	**Never versus sometimes**	**Never versus often**	**Never versus always**
**Mean difference**	−7.69914	−8.61758	−15.27143
**Significance**	0.016	0.022	0.001

## Discussion

Knowledge of the impact of MS on the person and the ability to assess symptom progression are critical to providing effective monitoring and evaluating DMTs [4]. Since the COVID-19 pandemic, many of the healthcare facilities encouraged the use of telephone-based assessment tools as an alternative way to follow up the MS patients, which will eventually decrease the risk of spread of such contagious disease by “staying safe at home.”

Most of the MS patients eventually experience walking difficulties. In this study, the Hauser Ambulation index was used to assess ambulation of the patient and hence the degree of disability. The EDSS scoring system remains a standard and well-established tool for assessing disability in MS patients. It is known that an EDSS score of 4 or more indicates that the patient has a degree of walking disabilities, or needs a walking aid or wheelchair [5], gaining over the concern that it is an ambulation-based measure. Since the EDSS is the main tool for the assessment of disability at our unit, we postulated that the Hauser Ambulation index can be a telephone-based alternative for assessing disability. The results showed that the Hauser Ambulation index was significantly correlated with the EDSS score done 1 month or less apart from the telephone call (*P*<0.001).

This supports that the Hauser Ambulation index can be a helpful tool for assessing ambulation in MS patients. It may be recommended to assess physical disability for MS patients. However, we would like to note the importance to monitor other disabilities in MS patients such as cognitive impairment which may be troublesome for many patients and may require symptomatic treatment.

The results of the MSNQ showed that one-third of the study population had evidence of cognitive and/or neuropsychological impairment. Although the MSNQ was not sensitive enough to detect cognitive impairment as the scores could be misled by the patient’s affect [6], we adopted it in this study because it is a relatively short and easy tool and can be self-reported, so can be implemented in mobile applications later on. Of course, these results had been correlated with other tools for assessing the cognitive functions and depression for confirming the credibility of this telephone-based tool, and this is a future scope for our center when those patients attend physically their next scheduled visits.

The lack of an association between the presence of cognitive and/or neuropsychological impairment (detected by MSNQ) and the clinical features of the disease (as disease duration, EDSS scoring, and type of MS) can be explained by the fact the cognitive impairment does not undergo a similar progression as physical disability [7].

In one study, the increase in the EDSS score did not predict deterioration in cognitive status [8]. Another study that compared patients with SPMS who did not have significantly greater disability than patients with RRMS reported a significant difference regarding cognitive impairment [7]. Moreover, some studies showed impaired neuropsychological performance in patients with clinically isolated syndrome and patients with no physical disability [9, 10].

This highlights the fact that physical disability and cognitive impairment are rather quite different features of MS that need to be monitored separately; in other words, cognitive functions cannot be judged based on the degree of physical disability. Physical disability seems to be related to the duration of disease, unlike cognitive impairment [7].

In this study, post hoc analysis regarding the cognitive and psychological impairment component of SMSS revealed that the patients who answered “Never” had significantly lower MSNQ scores compared to those who answered “Sometimes,” “Often,” and “Always,” which points that the cognitive and psychological impairment component of SMSS can be a reliable tool for a rough assessment of cognitive and psychological impairment in patients with MS.

On the contrary, the absence of significant difference after the comparison of the EDSS scores of the patients regarding the sensory-motor impairment component of SMSS may weaken its role as a rough tool for estimation of physical disability.

As it happened for many of the daily activities, the COVID-19 pandemic may have necessitated the engagement of alternative or complementary tools to monitor MS patients. The telephone-based assessments may be informative to the neurologist in monitoring MS patients especially if such information can be gathered effectively via reliable and valid standardized self-report questionnaires. One study investigated the modified telephone interview for cognitive status, a previously validated phone assessment for cognitive function in healthy elderly populations to detect mild cognitive impairment, in MS patients and demonstrated that a remotely administered cognitive assessment is quite feasible for conducting large epidemiologic studies in MS [11].

It is advised that assessments shall address the appearance of any new symptoms, walking disability, and cognitive functioning in order to have appropriate information about the patient’s status. The tools are recommended to be easily understood by the patient and if done via a telephone call shall not exceed 15 min as we noticed that most of the patients got bored from long telephone calls. Our future studies will implement strategies to assess the satisfaction of patients and physicians about such kind of assessments. We think about a mobile application that can include the assessment tools, which makes it easy for the patient to fulfill the items of the tools then being collected for analysis.

Lastly, it appears that remote monitoring is emerging as a new modality that can enable the neurologist to monitor their patients, and it is highly likely that we will continue to embrace the benefits of telemedicine and telephone-based assessments, and therefore, future directions shall include more evidence-based research on the diagnostic accuracy and reducing disparities in the access to telemedicine [12].

## Conclusion

The Hauser Ambulation index may be a reliable telephone-based tool for the assessment of physical disability. The MSNQ and the cognitive and psychological impairment component of SMSS can be used for the assessment of cognitive and psychological impairment among patients with MS. The telephone-based assessment tools may be helpful in monitoring MS patients provided that these tools aim to assess multiple scopes as ambulation, cognitive, and psychological impairment.

## Data Availability

Dataset is available as a master sheet in Excel format and publicly available in the Neurology Department, Ain Shams University, through communicating the corresponding author.

## Abbreviations

COVID-19: Coronavirus disease 2019
DMTs: Disease-modifying therapies
EDSS: Expanded Disability Status Scale
MS: Multiple sclerosis
MSNQ: Multiple Sclerosis Neuropsychology Questionnaire
PPMS: Primary progressive multiple sclerosis
RRMS: Relapsing-remitting multiple sclerosis
SMSS: Symptoms of Multiple Sclerosis Scale
SARS-CoV-2: Severe acute respiratory syndrome coronavirus 2
SPMS: Secondary progressive multiple sclerosis
